# A single point mutation causes one-way alteration of pheromone receptor function in two *Heliothis* species

**DOI:** 10.1016/j.isci.2021.102981

**Published:** 2021-08-16

**Authors:** Song Cao, Yang Liu, Bing Wang, Guirong Wang

**Affiliations:** 1 State Key Laboratory for Biology of Plant Diseases and Insect Pests, Institute of Plant Protection, Chinese Academy of Agricultural Sciences, Beijing 100193, China; 2 Shenzhen Branch, Guangdong Laboratory for Lingnan Modern Agriculture, Genome Analysis Laboratory of the Ministry of Agriculture and Rural Affairs, Agricultural Genomics Institute at Shenzhen, Chinese Academy of Agricultural Sciences, Shenzhen 518120, China

**Keywords:** Ecology, Biological sciences, Zoology, Neuroscience, Sensory neuroscience

## Abstract

The sex pheromone processing system of moths has been a major focus of research on olfaction and speciation, as it is highly specific and closely related to reproductive isolation. The two noctuid moths *Heliothis virescens* and *Heliothis subflexa* have been used as a model for deciphering the mechanisms underlying differentiation in pheromone communication, but no information exist regarding the functions of the pheromone receptors (PRs) of *H. subflexa*. Here, we functionally characterized all candidate PRs of *H. subflexa*, and found that only the response profile of OR6 differed between the two species*.* Through domain swapping and site-directed mutation followed by functional characterization, we identified a critical amino acid in OR6 caused a one-way alteration in specificity. This result suggests HsubOR6 evolved from an ancestral OR6 gene with a HvirOR6-like function and implies that the evolutionary direction of the receptor specificity was from the *H. virescens*-like pattern to *H. subflexa*-like pattern.

## Highlights

• Six pheromone receptors (PRs) were functionally characterized in *Heliothis subflexa*
• Function of one orthologous PR-OR6 differed between *H. subflexa* and *H. virescens*
• A critical amino acid residue caused one-way alteration of OR6 specificity
• This suggests HsubOR6 evolved from an ancestral gene with a HvirOR6-like function

## Graphical abstract

**Figure:**
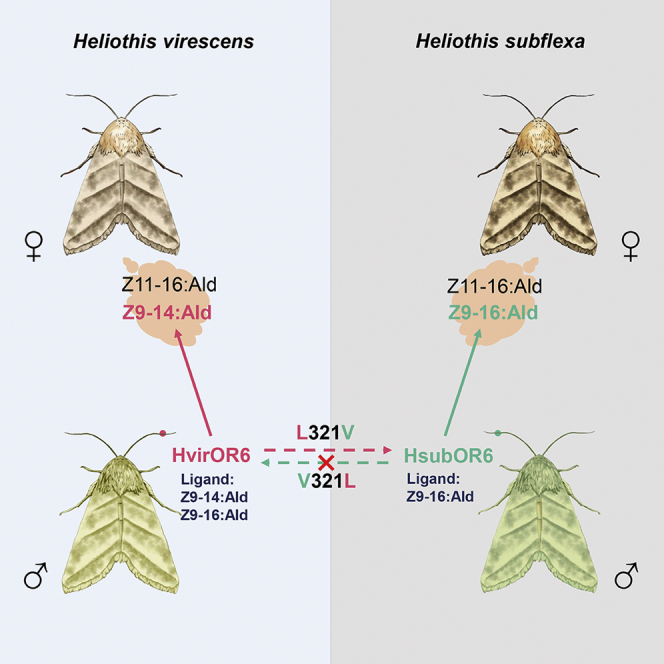

## Introduction

Most insects have a highly sensitive olfactory system, enabling adaptive behaviors in mating, hosts location, oviposition sites selection, and natural avoidance for enemies (Hildebrand, 1995; Masson and Mustaparta, 1990). The sex pheromone processing system of insects, especially moths, has become a focus of olfaction research, because sex pheromone reception in moths is not only highly sensitive and specific but also closely related to reproductive isolation (Birch et al., 1990; Ramaswamy, 1997; Allison and Cardé, 2016).

Sex pheromones play an essential role in species-specific reproductive communication in moths (Vickers, 2006; Cardé and Haynes, 2004). The composition and relative proportions of pheromone components are highly species-specific, and there is enormous diversity in pheromone composition even among closely related species. The sex pheromone perception system in moths is very complex, and enables efficient identification of mates as well as reproductive isolation, but its evolution is not well understood (Gould et al., 2009, 2010). Based on the typical roles of female moths as signal producers and male moths as signal receivers in the sex pheromone system, several evolutionary hypotheses, such as the stasis hypothesis, asymmetric tracking hypothesis, and wallflower hypothesis, have been proposed (Allison and Cardé, 2016; Cardé and Baker, 1984; Groot et al., 2006; Roelofs et al., 2002); however there is no consensus. This is mainly because existing studies show that the synthesis of sex pheromones in females and the perception of sex information in males are regulated by different genes and under independent genetic control (Gould et al., 2009, 2010; Löfstedt et al., 1989; Dopman et al., 2004; Unbehend et al., 2021). Although the contributions of male and female traits to the evolution of sex pheromone communication remain controversial, it is undeniable that male moths' perception of sex pheromone is one of the key steps.

The preference and sensitivity of male moths to female sex pheromones are controlled by olfactory circuitry, as reviewed in many previous works (Galizia and Rössler, 2010; Groot et al., 2016; Zhang et al., 2015; Stengl, 2010). The specific response of male moths to sex pheromones can be divided into two steps: first, at the peripheral nervous system level, the odor molecules are screened and perceived by olfactory receptor neurons (ORNs) and action potentials are generated with the involvement of diverse olfactory proteins; second, at the central nervous system level, the electrical signals are processed and induce a behavioral response (de Bruyne and Baker, 2008; Rützler and Zwiebel, 2005; Sato and Touhara, 2009; Leal, 2013). Differences in sex pheromone preferences are usually associated with changes in ORN function, which is determined by the OR expressed in it (Leal, 2013; Liu et al., 2013; Chang et al., 2016). Therefore, exploring the functions of pheromone receptors (PRs) and their differentiation between closely related species is essential to understanding the evolution of moth mating systems.

The noctuid moths *Heliothis virescens* and *Heliothis subflexa* (Lepidoptera: Noctuidae) are closely related moth species based on studies using morphological characters (Mitter et al., 1993), hybridization (Teal and Oostendorp, 1995a, 1995b; Vickers, 2006), and phylogenetic analyses using genetic markers (Cho et al., 1995, 2008; Fang et al., 1997). In both moths, Z11-16:Ald is the major component of the pheromone blend, which is essential for attraction behavior, but the types and ratios of minor components vary. In *H. virescens*, the secondary component Z9-14:Ald, whereas in *H. subflexa* the secondary components Z9-16:Ald, Z11-16:OH, and Z11-16:OAc are all necessary to produce attraction behavior in conspecific males, respectively (Groot et al., 2005; Vetter and Baker, 1983; Teal et al., 1981, 1986). Although the roles of all these components in moth behavior have not yet been unequivocally clarified, it is clear that the minor component Z9-14:Ald is necessary for attraction of *H. virescens* males, whereas *H. subflexa* males are only attracted when both Z9-16:Ald and Z11-16:OH are present (Vickers, 2002).

The mechanism of the differentiation in pheromone communication between *H. virescens* and *H. subflexa* was studied in a series of behavioral, physiological, and quantitative trait locus (QTL) analyses of hybrids and populations generated by backcrosses of the two species (Vickers, 2006; Gould et al., 2010; Baker et al., 2006). In a study that combined QTL analysis and candidate gene mapping, a small non-recombining region encoding four PRs, HvirOR6, HvirOR14, HvirOR15, and HvirOR16, was found to be associated with differences in male response to female pheromone blends between these two species (Gould et al., 2010). As the functions of PRs in *H. virescens* were successfully characterized (Grosse-Wilde et al., 2007; Krieger et al., 2009; Baker, 2009; Wang et al., 2011), HvirOR6, the receptor specific to Z9-14:Ald, was predicted to be involved in differential male responses to the secondary pheromone components Z9-16:Ald and Z9-14:Ald (Wang et al., 2011). However, without information about *H. subflexa* PR functions, it has not been possible to systematically study the functional differentiation in the PRs of *H. virescens* and *H. subflexa* and their roles in the speciation.

In this study, we cloned and functionally characterized six candidate PRs from *H. subflexa*. We found that only OR6 differentiated in function between *H. virescens* and *H. subflexa*. Through functional analysis of OR6 with swapped domains and a series of site-directed mutations, we identified a key site (position 321, L in HvirOR6, V in HsubOR6) that resulted in the functional changes between HvirOR6 and HsubOR6. Moreover, this change is direction dependent: a change in this amino acid in HvirOR6 changed the function of *H. virescens* to that of *H. subflexa*, but the reverse mutation did not change the function of *H. subflexa* to that of *H. virescens*.

## Results

### Phylogenetic analysis of the PRs of six heliothinae moths

**Figure 1:**
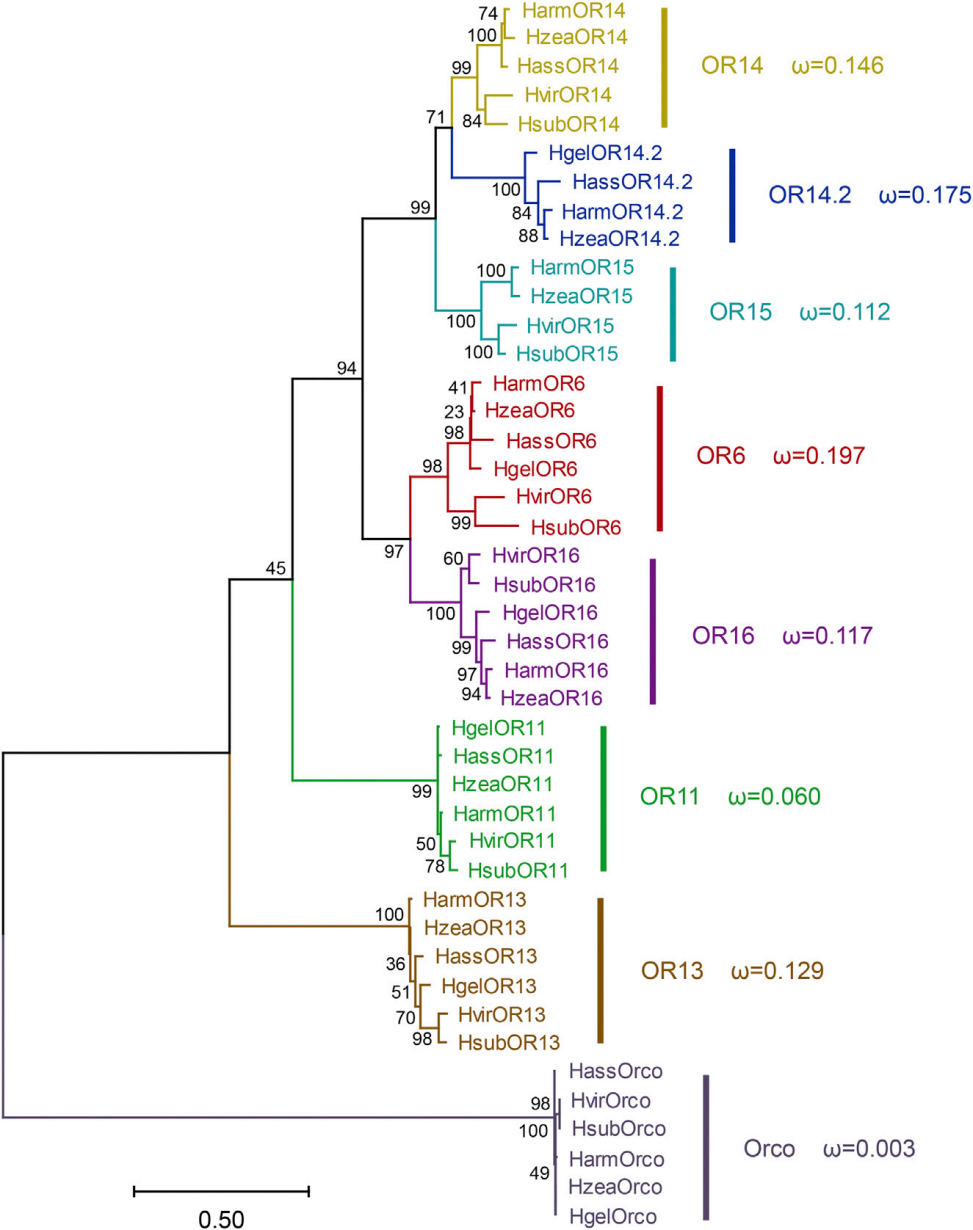
Phylogenetic tree of the PRs in Heliothinae moths The Orco lineage is defined as an outgroup. Bootstrap values are based on 1,000 replicates. The nonsynonymous (dN) to synonymous (dS) substitution ratio (ω) is provided for each clade.

Six full-length cDNAs encoding HsubPR were cloned using RT-PCR amplification. Multiple alignments of PR amino acid sequences from *H. subflexa* and *H. virescens* showed that the orthologous PRs in these two insects shared high sequence identities, ranging from 81.2% (OR6) to 96.5% (OR11) (Figure S1). Six odorant receptor co-receptor (Orco) genes and 37 PR genes from 6 Heliothinae moth species including 4 *Helicoverpa* species and 2 *Heliothis* species were used to construct a maximum likelihood phylogenetic tree (Figure 1). The 43 sequences formed 8 orthologous lineages: 7 Lepidoptera PR lineages and 1 conserved Orco lineage. To evaluate the evolutionary pressures acting on each of the eight clusters, we estimated the rates of nonsynonymous (dN) to synonymous (dS) substitutions (dN/dS or ω) in each lineage. The ω values of Orco and all the orthologous PRs were much smaller than 1, indicating that these ORs are under strong purifying selection. The ω for the Orco orthologs was the lowest (ω = 0.003) as expected, given the high functional conservation of Orco genes (Vosshall and Hansson, 2011). In contrast, the ω for OR6 orthologs was the highest (ω = 0.197), which suggests that this OR is more likely to have undergone functional differentiation in these species (Figure 1).

### OR6 from *H. subflexa* and *H. virescens* exhibited different response profile to Z9-16:Ald and Z9-14:Ald

**Figure 2:**
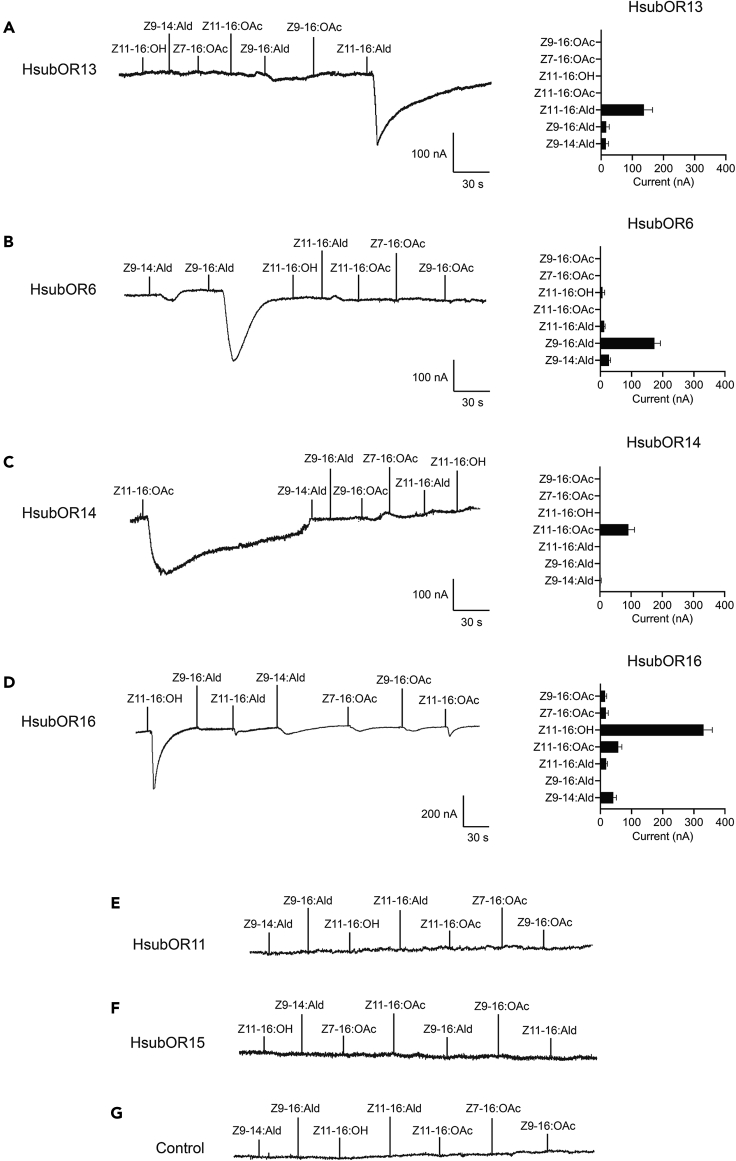
Inward current responses (left) and response profiles (right) of *Xenopus* oocytes co-expressing HsubPR/HsubOrco and treated with a 10^−4^ M solution of pheromone compounds (A) HsubOR13/HsubOrco. (B) HsubOR6/HsubOrco. (C) HsubOR14/HsubOrco. (D) HsubOR16/HsubOrco. (E) HsubOR11/HsubOrco. (F) HsubOR15/HsubOrco. (G) Buffer-injected *Xenopus* oocytes failed to respond to any of the pheromone stimuli. Error bars indicate SEM (*n* = 4 to 9).

To decipher the ligands for each PR, all PRs in *H. subflexa* were individually co-expressed with HsubOrco in *Xenopus* oocytes followed by testing of the electrophysiological responses elicited by a panel of *H. subflexa* and *H. virescens* pheromone compounds. The oocytes co-expressing HsubOR13 and HsubOrco specifically responded to the major sex pheromone component Z11-16:Ald (Figure 2A). The *Xenopus* oocytes co-expressing HsubOR6 and HsubOrco responded to the secondary sex pheromone component Z9-16:Ald and showed little if any response to Z9-14:Ald (Figure 2B). HsubOR14 was specifically activated by Z11-16:OAc (Figure 2C), and HsubOR16 responded robustly to Z11-16:OH (Figure 2D). The other two candidate pheromone receptors HsubOR11 and HsubOR15 failed to respond to any of the candidate pheromone compounds tested in this study (Figures 2E and 2F). Buffer-injected control oocytes were also unresponsive to any of the seven compounds (Figure 2G). HsubORs showed highly specific responses to the pheromones tested, and most HsubORs exhibited pheromone sensitivities similar to those of HvirORs.

**Figure 3:**
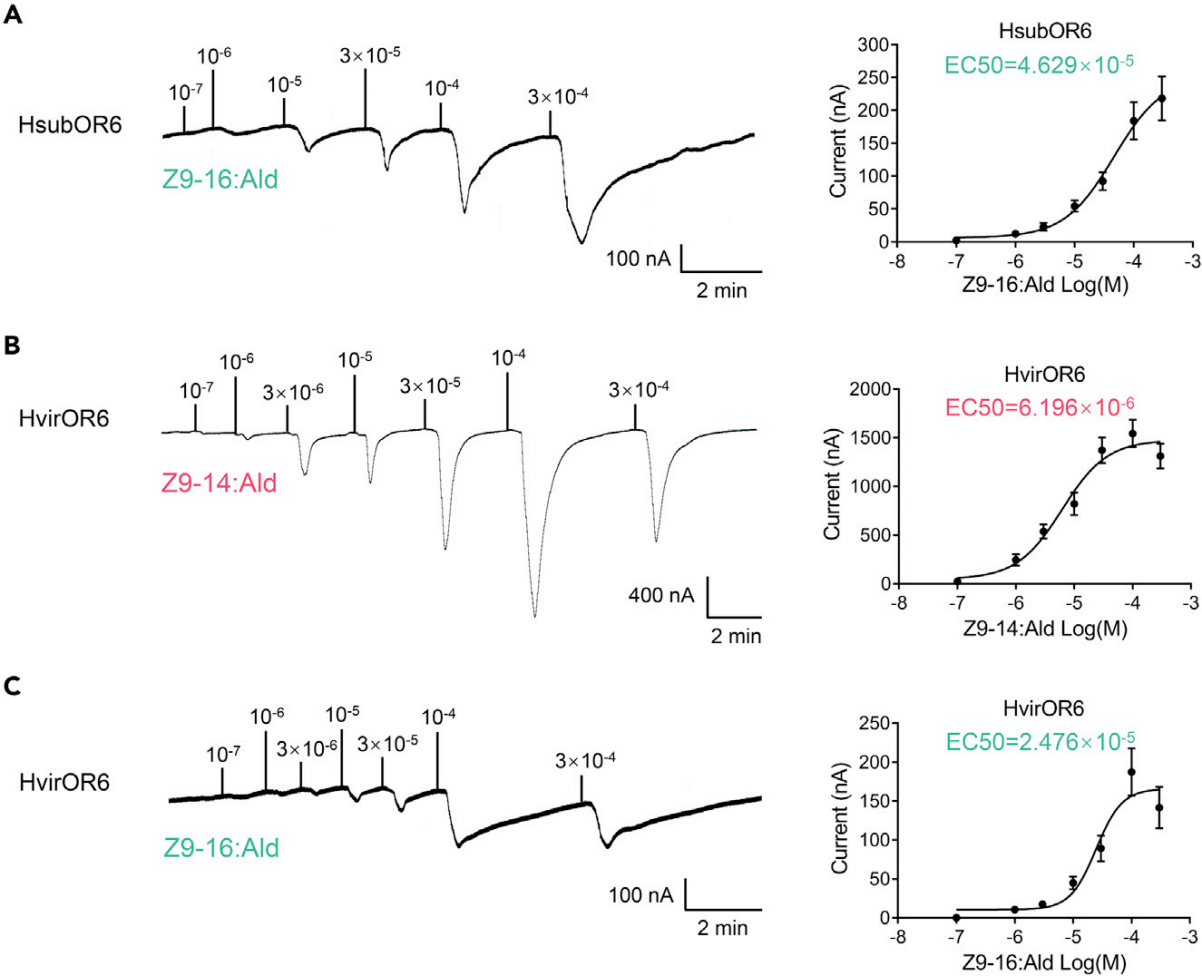
The sensitivity of HsubOR6 and HvirOR6 to Z9-16:Ald and Z9-14:Ald (A) (Left) HsubOR6/HsubOrco *Xenopus* oocytes stimulated with a range of Z9-16:Ald concentrations. (Right) Dose–response curve for HsubOR6/HsubOrco *Xenopus* oocytes treated with Z9-16:Ald. EC50 = 4.629 × 10^−5^ M. (B) (Left) HvirOR6/HvirOrco *Xenopus* oocytes stimulated with a range of Z9-14:Ald concentrations. (Right) Dose–response curve for HvirOR6/HvirOrco *Xenopus* oocytes treated with Z9-14:Ald. EC50 = 6.196 × 10^−6^ M. (C) (Left) HvirOR6/HvirOrco *Xenopus* oocytes stimulated with a range of Z9-16:Ald concentrations. (Right) Dose–response curve for HvirOR6/HvirOrco *Xenopus* oocytes treated with Z9-16:Ald. EC50 = 2.476 × 10^−5^ M. Error bars indicate SEM (*n* = 7 to 10).

Of the six pairs of orthologous PRs, only the OR6 pair was found to be functionally differentiated between *H. virescens* and *H. subflexa.* Considering the evidence of purifying selection acting on OR6 (Figure 1), we compared the functions of the two orthologous OR6 *in vitro*. HsubOR6 was specifically responsive to Z9-16:Ald, with an average response of 184.04 nA at a concentration of 10^−4^ M. In dose-response studies, 10^−6^ M Z9-16:Ald could elicit significant responses from oocytes that co-expressed HsubOR6 and Orco with an EC50 value of 4.629 × 10^−5^ M (Figure 3A). In contrast, HvirOR6 was responsive to both Z9-14:Ald (EC50 value of 6.196 × 10^−6^ M) and Z9-16:Ald (EC50 value 6.196 × 10^−6^ M), with a specifically higher response to Z9-14:Ald, at a concentration of 10^−4^ M; the response of HvirOR6 to Z9-14:Ald was 1,541.96 nA, about 8.23 times higher than that to Z9-16:Ald (187.44 nA) (Figures 3B and 3C). These results showed that HsubOR6 lost its ability to recognize Z9-14:Ald.

### Chimeric genes construction and functional analysis

**Figure 4:**
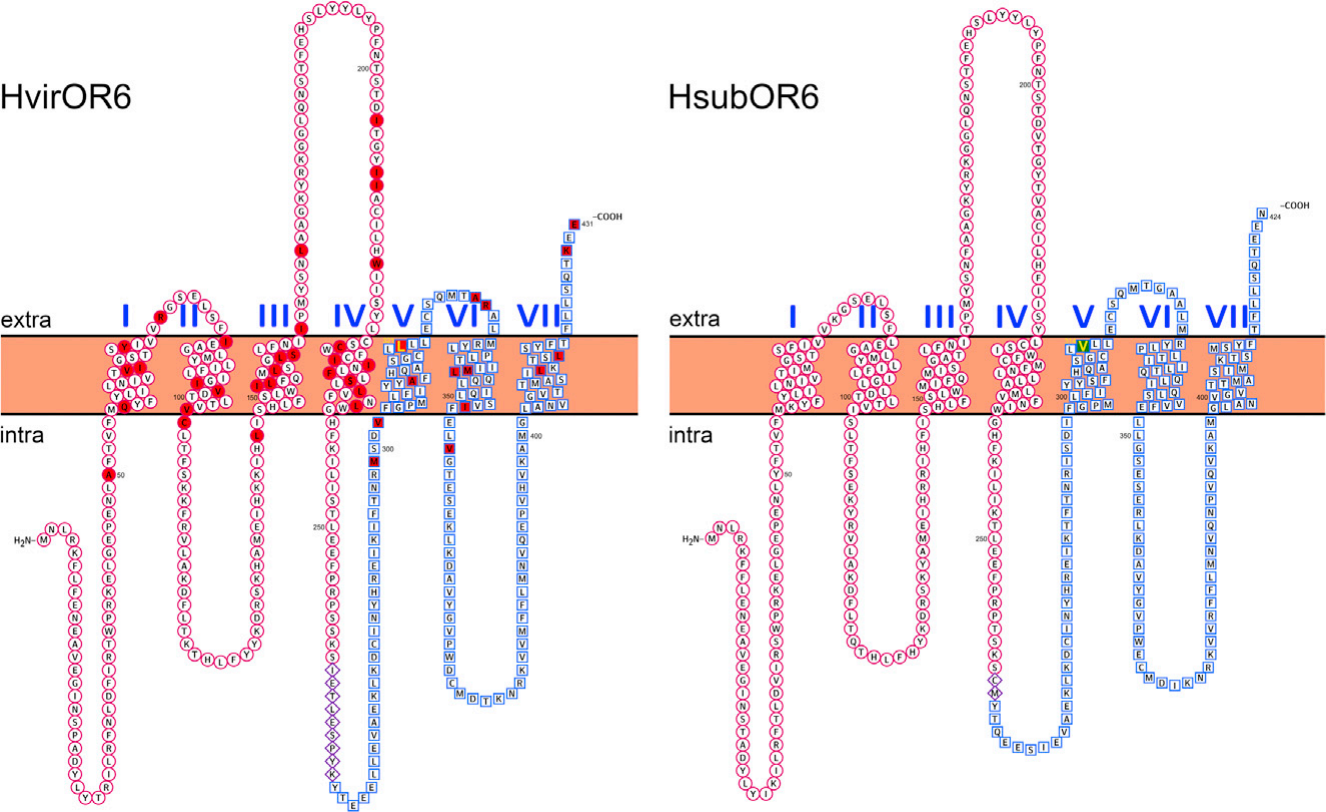
Comparison of the amino acid sequences and transmembrane structures of HvirOR6 (left) and HsubOR6 (right) Region 1 is marked by a red circle. Region 2 is marked by a purple diamond. Region 3 is marked by a blue square. The mutated sites in HvirOR6 and HsubOR6 are labeled in red and green, respectively. The key sites in HvirOR6 (L) and HsubOR6 (V) are highlighted in yellow.

HvirOR6 and HsubOR6 contain 431 and 424 amino acids, respectively, and they share 81.21% amino acid identity; 72 amino acids differ and there is one insertion-deletion of 9 amino acids in the intracellular region between the 4^th^ and 5^th^ transmembrane domains (TMDs) (Figures 4; S2). To investigate which amino acids determine the specificity to Z9-14:Ald and Z9-16:Ald, we divided the sequence into three regions and tested six chimeric genes with different combinations of these regions (Figure S2B).

**Figure 5:**
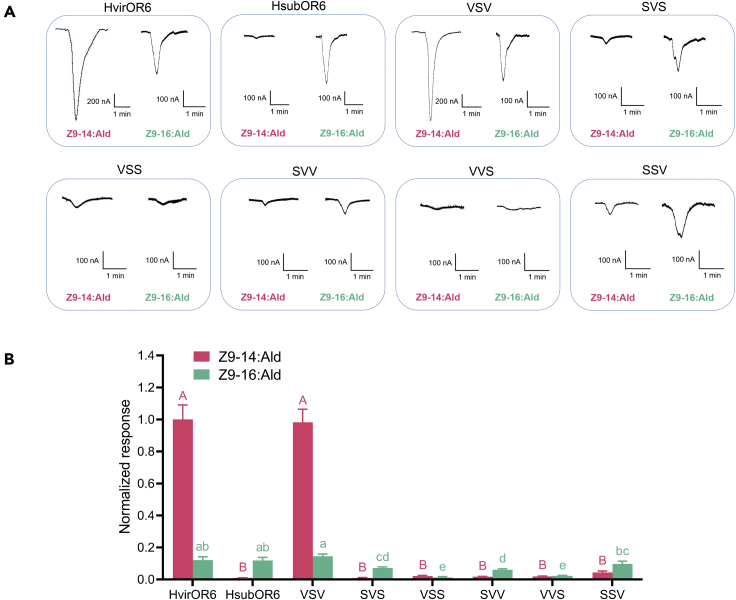
Comparison of the responses of six chimeric proteins to 10^−4^ M Z9-16:Ald and Z9-14:Ald (A) Inward currents generated in oocytes co-expressing HvirOrco and one of six chimeric genes in response to 10^−4^ M Z9-16:Ald and Z9-14:Ald. (B) Response profiles for oocytes co-expressing HvirOrco and one of six chimeric genes treated with 10^−4^ M Z9-16:Ald and Z9-14:Ald. Error bars indicate SEM (*n* = 7 to 10).

Functional analysis of the chimeric genes showed that region 2 of OR6 (V2 and S2) has no significant influence on the response to Z9-14:Ald or Z9-16:Ald (Figure 5). If we replaced the sequence from one species with that of the other species, the responses of the chimeric OR6s (VSV and SVS) to Z9-14:Ald did not change significantly compared with those of the original sequences (VVV and SSS) (Figure 5). The responses of the other four chimeric OR6s (VSS, SVV, VVS, and SSV) to Z9-14:Ald and Z9-16:Ald decreased significantly compared with those of the original sequences (Figure 5). These results suggested the first and the third regions of OR6 are both essential for binding Z9-14:Ald and Z9-16:Ald.

### A single point mutation changes the specificity of HvirOR6 to that of HsubOR6

**Figure 6:**
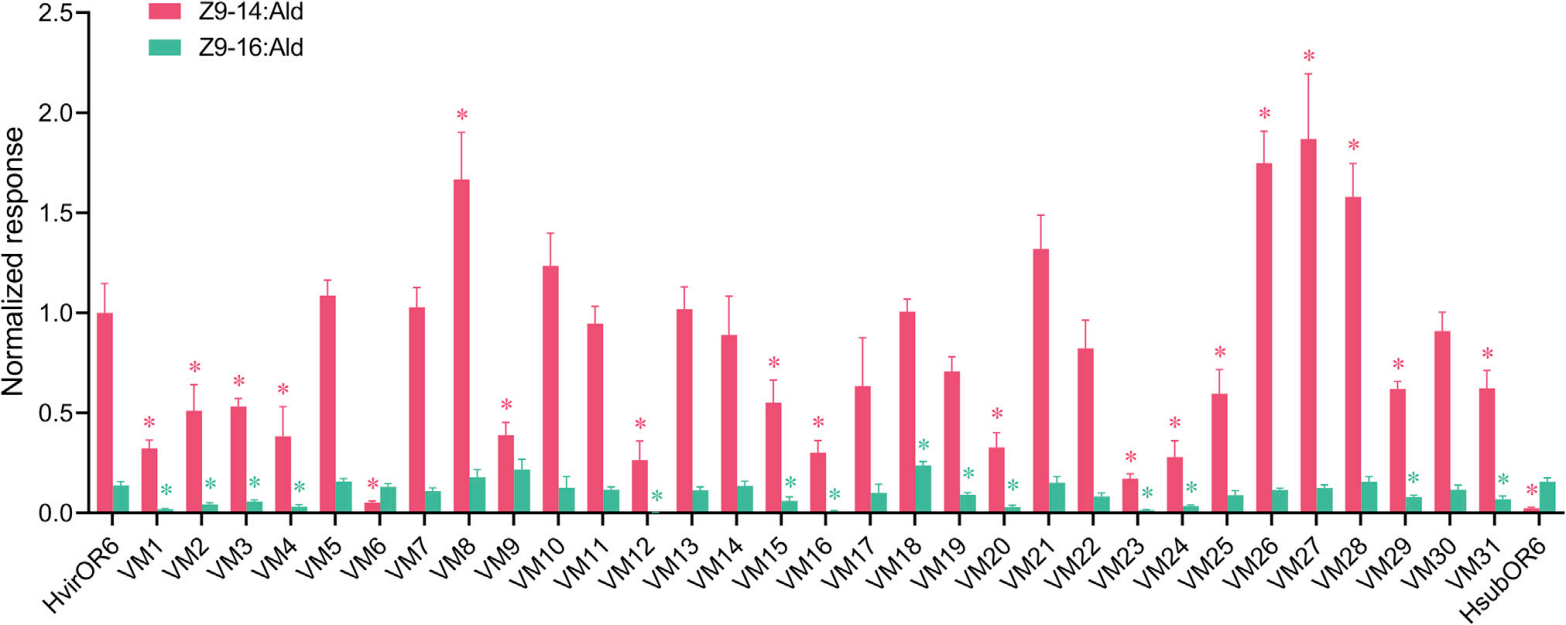
Response profiles of HvirOR6 mutants. Response profiles of mutated HvirOR6 with amino acid sites matching those in HsubOR6 to 10^−4^ M Z9-16:Ald and Z9-14:Ald. The asterisk indicates a significant difference (*P* < 0.05); error bars indicate SEM (*n* = 6 to 10).

Besides the differences in region 2 of OR6, HsubOR6 and HvirOR6 differ at 72 amino acid sites (Figures 4; S2). To elucidate the molecular mechanism underlying the difference in pheromone binding specificity, we conducted site-directed mutagenesis experiments to determine the key residues involved in pheromone binding. The amino acid sites in the TMD and extracellular loop (ECL) were previously predicted to form the ligand-binding pocket and determine the function of ORs (Guo and Kim, 2010; Nichols and Luetje, 2010; Pellegrino et al., 2011; Leary et al., 2012; Hughes et al., 2014; Rahman and Luetje, 2017; Yang et al., 2017). Therefore, we mutated a total of 44 amino acid residues in HvirOR6 (27 residues in the TMD, 11 residues in the ECL, and 6 residues in the intracellular region close to the TMD), generating 31 mutant versions of HvirOR6 containing the HsubOR6 sequence (VM1–VM31); detailed information is provided in Figure 4 and Table S1 in supplemental information. Functional analysis of the 31 mutants showed that 10 mutants (VM5, VM7, VM10, VM11, VM13, VM14, VM17, VM21, VM22, and VM30) did not have altered responses to either Z9-14:Ald or Z9-16:Ald. But in the remaining 21 mutants, the function of OR6 changed, which suggested that several sites are critical to the function of HvirOR6. Twelve mutants (VM1, VM2, VM3, VM4, VM12, VM15, VM16, VM20, VM23, VM24, VM29, and VM31) resulted in a significant decrease in the response to both Z9-14:Ald and Z9-16:Ald. Four mutants (VM8, VM26, VM27, and VM28) had increased responses to Z9-14:Ald, but the mutants VM9 and VM25 had decreased responses to Z9-14:Ald (Figure 6). In addition, two mutants (VM18 and VM19) only had altered responses to Z9-16:Ald, with increased response for VM18 and decreased response for VM19. Of special interest, one site-directed mutation (L321V) in HvirOR6 (VM6) resulted in the function completely changing to that of HsubOR6: the mutant lost its response to Z9-14:Ald while retaining the response to Z9-16:Ald (Figure 6).

**Figure 7:**
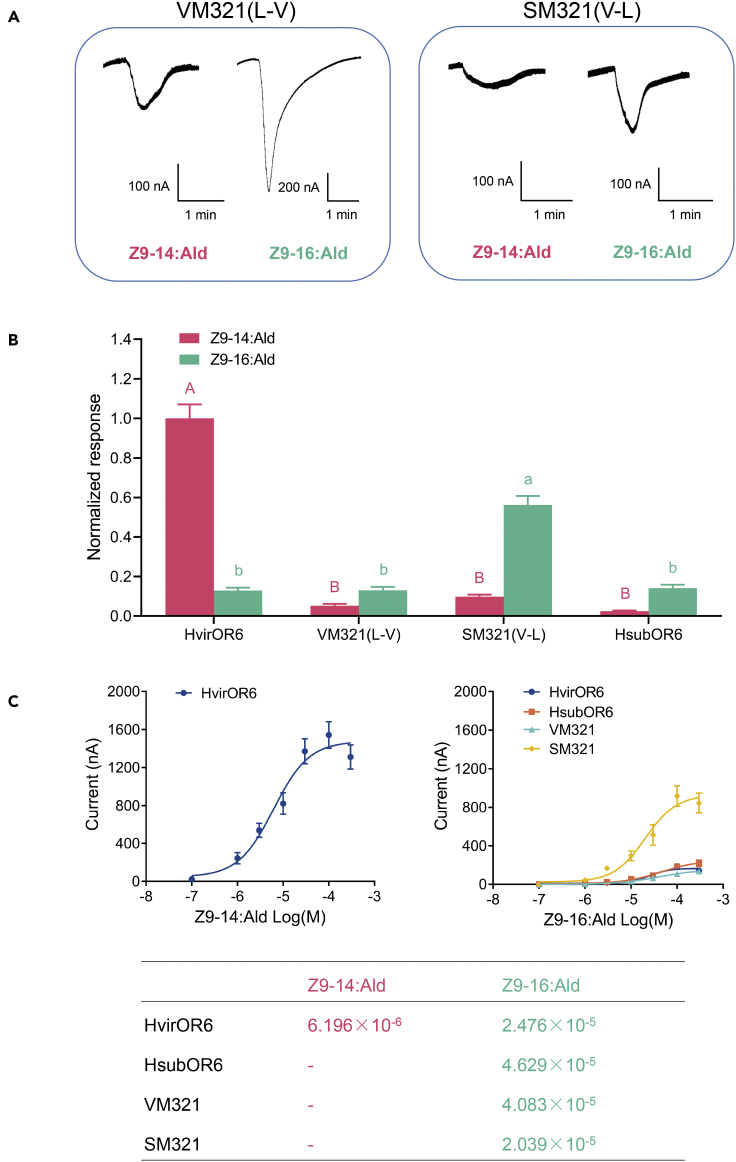
Comparison of the responses of OR6 with mutation of a key site to Z9-16:Ald and Z9-14:Ald (A) Inward current responses of oocytes co-expressing HvirOrco and one of two mutated OR6 to 10^−4^ M Z9-16:Ald and Z9-14:Ald. (B) Response profiles of oocytes co-expressing HvirOrco and one of two mutated OR6 to 10^−4^ M Z9-16:Ald and Z9-14:Ald. Error bars indicate SEM (*n* = 13 to 23). (C) (Upper) Dose–response curves for HvirOR6, HsubOR6, and their mutants co-expressed with Orco and treated with Z9-14:Ald and Z9-16:Ald (*n* = 5 to 13). (Lower) Comparison of the EC50 values of HvirOR6/HsubOR6/VM321/SM321 for Z9-14:Ald and Z9-16:Ald.

As site 321 was demonstrated to be the key site involved in the functional transformation between HvirOR6 and HsubOR6, we further compared the sensitivity of the mutant L321V (VM321) to Z9-14:Ald and Z9-16:Ald with those of HvirOR6 and HsubOR6. The results showed that the response profile of VM321 was exactly the same as that of HsubOR6 (Figures 7A and 7B). VM321 lost its ability to bind Z9-14:Ald, and its EC50 for Z9-16:Ald was 4.083 × 10^−5^ M, which was similar to that of HsubOR6 (4.629 × 10^−5^ M) (Figure 7C).

### Mutation in a single amino acid is not sufficient to change the function of HsubOR6 to that of HvirOR6

As one site-directed mutation (VM321) led to a complete change in the function of HvirOR6 to that of HsubOR6, we next mutated this site (V321L) in HsubOR6 (SM321) to clarify whether a change in this site could also lead to a change in the function of HsubOR6 to that of HvirOR6. SM321 did not change the function of HsubOR6, as the response of SM321 to Z9-16:Ald increased, whereas, the response to Z9-14:Ald remained unchanged (Figures 7A and 7B). We next compared the EC50 of SM321 with those of HvirOR6 and HsubOR6, and its sensitivity to Z9-16:Ald (2.039 × 10^−5^ M) was similar to those of HvirOR6 (2.476 × 10^−5^ M) and HsubOR6 (4.629 × 10^−5^ M) (Figure 7C). These results confirmed that the mutation of one site, 321, could cause a transformation of the function of HvirOR6 to that of HsubOR6, but the reverse mutation did not lead to HsubOR6 gaining the function of HvirOR6, although this mutation resulted in a change in the absolute response value. We hypothesize that the change in function from HsubOR6 to HvirOR6 requires additional site changes.

## Discussion

The two closely related noctuid species *H. virescens* and *H. subflexa* are generally accepted as ideal models to study the mechanisms underlying the differentiation in pheromone communication between moth species (Mitter et al., 1993). Typical differences in the sex pheromone communication systems of these two species are the secondary sex pheromone components (Z9-14:Ald or Z9-16:Ald) and corresponding sensory systems (Groot et al., 2005; Vetter and Baker, 1983; Teal et al., 1981, 1986). Differences in the function of ORNs corresponding to secondary components between *H. virescens* and *H. subflexa* suggest that there is a difference in receptor function (Baker et al., 2004). Results from previous genome mapping studies and QTL analysis helped us to identify differential four PR candidates, namely, HvirOR6, HvirOR14, HvirOR15, and HvirOR16 (Gould et al., 2010). Further functional studies of HvirORs demonstrated that OR6 is the receptor that has undergone functional differentiation, as HvirOR6 is the only PR responding to Z9-14:Ald and Z9-16:Ald (Wang et al., 2011). In this study, we first studied the function of PRs in *H. subflexa* to make it possible to compare the functions of orthologous PRs in *H. virescens* and *H. subflexa*. We found that four *H. subflexa* PRs (HsubOR6, HsubOR13, HsubOR14, and HsubOR16) have different sex pheromones as ligands (Figure 2). Notably, only one PR showed functional differences from its HvirOR ortholog. HsubOR6 lost its ability to recognize Z9-14:Ald and only responds to Z9-16:Ald (Figures 2 and 3). This result is consistent with the results of evolutionary analysis, which indicated that purifying selection pressure on HsubOR6 is the highest among all seven orthologous PRs (Figure 1). The functional differentiation of OR6 between *H. virescens* and *H. subflexa* is the fundamental reason for the difference in the perception of secondary pheromones between the two species.

This functional differentiation also makes *H. virescens* and *H. subflexa* ideal models to study the structure-function relationships of PRs. Researchers studying such relationships for other ORs have predicted that key amino acids in the TMD and ECL form the ligand-binding pocket and determine the function of the OR (Guo and Kim, 2010). This hypothesis has been confirmed by relevant studies in insects. Nichols and Luetje showed that TMD3 of OR85b in *Drosophila melanogaster* is essential for ligand-receptor interactions (Nichols and Luetje, 2010). Pellegrino et al. showed that polymorphism of a site in TMD3 of OR59b in *D. melanogaster* altered the sensitivity to DEET (Pellegrino et al., 2011). In *Ostrinia furnacalis* and *Ostrinia nubilalis*, a single amino acid mutation in TMD3 could change the ligand specificity of OR3 (Leary et al., 2012). Hughes et al. reported that a single mutation in the ECL2-TMD4 interface of OR15 altered the responses to odors in *Anopheles gambiae* (Hughes et al., 2014; Rahman and Luetje, 2017). Two single point mutations in TMD4 and TMD6 were found to shift the ligand selectivity of a pheromone receptor OR14b in the Noctuidae moths *Helicoverpa armigera* and the *Helicoverpa assulta* (Yang et al., 2017).

The functional differentiation between HvirOR6 and HsubOR6 provides a new opportunity to study the relationship between receptor function and structure. The biggest difference between the two sequences is a 9 amino acid insertion-deletion located in intracellular domain 3 (ICD3). However, we found that this region had no significant effect on the function of OR6, thus demonstrating that it is not critical in determining the specificity of ORs. Based on the results of mutation studies and further functional validation of single-site mutants, we identified a key site in TMD5 (position 321, L in HvirOR6, and V in HsubOR6) causing functional changes between HvirOR6 and HsubOR6. As in HvirOR6, the mutant VM321 lost its response to Z9-14:Ald. The experimental results indicated that the mutation of many sites could lead to changes in HvirOR6 function, especially the loss of the response to Z9-14:Ald (Figure 6). However, we found substitution of this site in HsubOR6 with the amino acid found in HvirOR6 (V321L) did not lead to a change in function to that of HvirOR6. We speculate that gain of function in an OR requires the involvement of multiple sites.

The noctuid moths *H. virescens* and *H. subflexa* are closely related species, which are separated by about 2.5 Mya (Mitter et al., 1993; Fang et al., 1997; Cho et al., 2008). Although not considered to be sister species, these two species and other species in the “*Heliothis* group” are considered to be an ideal model for studying the relationship between species evolution and changes in feeding habit (Cho et al., 1995, 2008; Groot et al., 2011). A classical concept holds the point that the host plant specialization is a dead-end in insect evolution (Mayr, 1963), which is consistent with the situation reported in heliothine moths that diverge before *Heliothis* (Cho et al., 2008). It is well-known that *H. virescens* is polyphagous and *H. subflexa* is oligophagous. Thus, if the theory of evolution is also suitable to *Heliothis* moths, we would like to speculate that *H. virescens* diverged earlier than *H. subflexa*, which further supports our implication that the evolutionary direction of OR6 was from the *H. virescens* function to the *H. subflexa* function. But in the *H. virescens* subgroup, host plant records for the members other than *H. virescens* and *H. subflexa* are insufficient, and there is no definite conclusion about whether their common ancestor was a monophagous species like *H. subflexa* or a polyphagous species like *H. virescens* (Cho et al., 2008). Groot et al. (2011) conducted a comparative population genetic analysis of *H. virescens* and *H. subflexa*, and showed that *H. subflexa* has more population differentiation than *H. virescens*, presuming the host specialization is a dynamic trait rather than an evolutionary dead end (Groot et al., 2011). Because the evolution of insects is directly related to the production and response to sex pheromones, our research on the mechanism of the differentiation of sex pheromone perception in *H. virescens* and *H. subflexa* is helpful for explaining species differentiation in the *H. virescens* subgroup. We found that the change of one amino acid site in OR6 could cause a functional change from *H. virescens* to *H. subflexa*, but the reverse mutation could not cause change the function of *H. subflexa* to that of *H. virescens*, implying that the evolutionary direction was from the *H. virescens* function to the *H. subflexa* function.

### Limitation of the study

This work indicated that one amino acid shift in HvirOR6 could result in a functional change to HsubOR6, but the key residues that are responsible for the functional shift of OR6 from *H. subflexa* to *H. virescens* have not been identified. Therefore, we are not able to identify the complete amino acid sites composition involved in Z9-14:Ald recognition in HvirOR6. Moreover, there is still a long way to clarify the relationship between the sequence and function of OR6.

## STAR★Methods

### Key resources table

**Table:** 

REAGENT or RESOURCE	SOURCE	IDENTIFIER
**Chemicals**		

(*Z*)-11-hexadecenal	Changzhou Nimrod Inc.	P5114; CAS: 53939-28-9
(*Z*)-9-tetradecenal	Changzhou Nimrod Inc.	95126; CAS: 53939-27-8
(*Z*)-9-hexadecenal	Changzhou Nimrod Inc.	P5111; CAS: 56219-04-6
(*Z*)-11-hexadecenyl acetate	Changzhou Nimrod Inc.	P5318; CAS: 34010-21-4
(*Z*)-9-hexadecenyl acetate	Changzhou Nimrod Inc.	P5320; CAS: 34010-20-3
(*Z*)-7-hexadecenyl acetate	Changzhou Nimrod Inc.	P5351; CAS: 23192-42-9
(*Z*)-11-hexadecen-1-ol	Changzhou Nimrod Inc.	P5214; CAS: 56683-54-6

**Deposited data**		

HsubOrco	This study	GenBank: MN399805
HsubOR6	This study	GenBank: MN399806
HsubOR11	This study	GenBank: MN399807
HsubOR13	This study	GenBank: MN399808
HsubOR14	This study	GenBank: MN399809
HsubOR15	This study	GenBank: MN399810
HsubOR16	This study	GenBank: MN399811

**Oligonucleotides**		

For information regarding oligonucleotide sequences used in this study please refer to Table S1	This study	NA

**Software and algorithms**		

GraphPad Prism 8.0	GraphPad Software	https://www.graphpad.com
SAS v8	SAS	https://www.sas.com/
PAML 4.9	(Yang, 2007)	http://abacus.gene.ucl.ac.uk/software/paml.html
MEGA Ⅹ	(Kumar et al., 2018)	https://www.megasoftware.net/

### Resource availability

#### Lead contact

Further information and requests should be directed to the Lead Contact, Guirong Wang (wangguirong@caas.cn).

#### Materials availability

This study did not generate new unique reagents. All the materials and methods used for the generation of data and analysis are mentioned in the manuscript.

### Experimental model and subject details

2 years old mature female *Xenopus laevis* frogs were purchased from *Xenopus* Resource Center (Qingdao, China). All animals were maintained in a recirculating tank system at a temperature of 18–20°C. Frogs were fed with adult *Xenopus* irradiated diet (Zeigler, Prod. No. 316518-18-2412). All dissection procedures are performed under anesthesia. The protocols used in our work were approved by the Institutional Animal Care and Use Committee of Chinese Academy of Agricultural Sciences, Beijing, China.

### Method details

#### Pheromone components

(*Z*)-11-hexadecenal (Z11-16:Ald), (*Z*)-9-tetradecenal (Z9-14:Ald), (*Z*)-9-hexadecenal (Z9-16:Ald), (*Z*)-11-hexadecenyl acetate (Z11-16:OAc), (*Z*)-9-hexadecenyl acetate (Z9-16:OAc), (*Z*)-7-hexadecenyl acetate (Z7-16:OAc), and (*Z*)-11-hexadecen-1-ol (Z11-16:OH) were purchased from Nimrod Inc. (Changzhou, China), all with minimum purity of 95%. Stock solutions (1 M) were dissolved in dimethyl sulfoxide (DMSO) and stored at −20°C. Before experiments, the stock solution was diluted in 1 × Ringer's buffer (96 mM NaCl, 2 mM KCl, 5 mM MgCl_2_, 0.8 mM CaCl_2_ and 5 mM HEPES, pH 7.6) to working concentration of 10^−4^ M. 1 × Ringer's buffer containing 0.1% DMSO was used as a negative control.

#### Transcript sequence and gene clone

*H. subflexa* were obtained from a laboratory culture maintained at North Carolina State University. The sequences of the 6 PR and 1 Orco genes in *H. subflexa* were obtained based on an analysis of the cDNA library of newly enclosed male *H. subflexa*. Total RNA from a pool of five adult male antennae was extracted using QIAGEN RNeasy Plus kit. cDNA was synthesized using QIAGEN QuantiTect Reverse Transcription kit with a total RNA concentration of 0.5 μg. Primers were designed to amplify the full length of the coding region sequence for each of the genes (Table S1). PCR was performed using the FastStart High-Fidelity PCR system (Roche, Indianapolis, IN, USA) under the following conditions: 94°C for 3 min, followed by 19 cycles of denaturation at 94°C for 1 min, annealing at 57°C for 1 min with 0.5°C decreasing per cycle, and extension at 72°C for 2 min followed by 19 cycles of denaturation at 94°C for 1 min, annealing at 47°C for 1 min and extension at 72°C for 2 min, followed by 72°C for 7 min. PCR products were purified and sequenced by Genewiz (South Plainfield, NJ, USA). Sequences have been deposited and can be found in GenBank (GenBank: MN399805- MN399811).

The PR and Orco sequences of *H. virescens*, *H. armigera* and *H. assulta* were obtained from previous reports. The PR and Orco sequences of *H. zea* and *H. gelotopoeon* were obtained based on the analysis of antennal transcriptome (Yang Liu, unpublished). All the candidate PR and Orco sequences were further confirmed by RACE and PCR amplification.

#### Vector construction

The full-length cDNA sequences of 6 HsubPRs (*HsubOR6*, *HsubOR11*, *HsubOR13*, *HsubOR14*, *HsubOR15* and *HsubOR16*), *HvirOR6*, *HsubOrco* and *HvirOrco* were first cloned into pENTR/D-TOPO entry vectors (Invitrogen, Carlsbad, CA, USA) and then subcloned into pSP64T (converted from pSP64T-Oligo) destination vectors by means of the Gateway LR reaction. The primers used for vector constructed were listed in Table S1 in supplemental information.

#### Sequences and phylogenetic analyses

The ORFs (Open reading frames) of all PR and Orco genes were predicted by using ExPASy (Expert Protein Analysis System) server version (http://web.expasy.org/translate/). The amino acid sequences of 6 PRs in *H. virescens* and 6 PRs in *H. subflexa* were aligned using DNAMAN software (version 8). The TMDs (transmembrane domains) were predicted using TMHMM server version 2.0 (http://www.cbs.dtu.dk/services/TMHMM/). The 43 OR amino acid sequences used to construct a phylogenetic tree were aligned by using MUSCLE software. The maximum likelihood method was employed to construct the phylogenetic tree by MEGA X software with Jones-Taylor-Thornton amino acid substitution model (JTT). Bootstrap analysis was performed base on 1,000 replicates.

Tests of selection were performed using the codeml procedure implemented in the PAML 4.9 package that estimates ratios of the normalized nonsynonymous (dN) to synonymous (dS) substitution rate (ω) by the maximum likelihood method (where ω > 1 is considered evidence of positive selection and ω < 1 evidence of purifying selection). Considering functional divergence among gene paralogs or species orthologues, the selective pressures may vary among branches in the tree. So the selective pressures of 8 OR orthologs (Orco, OR6, OR11, OR13, OR14, OR14.2, OR15 and OR16) in these 6 species were tested separately. For each lineage, the codon sequences were aligned using MUSCLE-Code and a maximum likelihood phylogenetic tree was reconstructed with Maximum Composite Likelihood model by MEGA X software. The substitution rate (ω) of each lineage was calculated in codeml procedure with Site model Model 0: one-ratio.

#### Construction of chimeric OR6 sequences

According to the sequence alignment of HvirOR6 and HsubOR6, we can divide the OR6 amino acid sequence of the two species into three fragments. Fragment 1 of the two species (V1 and S1) contains 260 amino acids (aa). Fragment 2 is species-specific and it contains 9 aa or 2 aa in HvirOR6 (V2) or HsubOR6 (S2) separately (Figure S2). Fragment 3 contains 162 amino acids (aa). To clarify the relationship between structure and function in the two orthologous HvirOR6 and HsubOR6, we constructed 6 chimeric OR6 sequences (VSV, SVS, VSS, SVV, VVS and SSV) compared with HvirOR6 (VVV) and HsubOR6 (SSS) using the two steps Overlap PCR (Figure S2). In the first step, two fragments of each chimeric sequence were amplified using a HvirOR6 or HsubOR6 specific primer and a chimeric gene specific primer with HvirOR6 or HsubOR6 as templates. The PCR conditions were: 95°C for 3 min, then 35 cycles of 95°C for 30 s, 55°C for 45 s, 72°C for 1 min, and final elongation 72°C for 10 min. The PCR products were excised from gel and extracted. In the second step, the two fragments which have overlap region were used as the template. The whole chimeric OR6 sequences were amplified using HvirOR6 or HsubOR6 specific primers. The PCR conditions were: 95°C for 3 min, then 25 cycles of 95°C for 30 s, 53°C for 45 s, 72°C for 2 min, and final elongation 72°C for 10 min. The 6 chimeric OR6 sequences were then subcloned into pSP64T vectors as described above. The primers used for chimeric sequences construction were listed Table S1 in supplemental information.

#### Preparation of site-directed mutants

The site-directed mutants were generated into the HvirOR6 and HsubOR6 pSP64T vectors using the Phusion Site-Directed Mutagenesis Kit (Thermo Scientific, Waltham, MA, USA) following the manufacturer's instruction. A pair of phosphorylated primers contained the mutation sites were designed and used for amplifying each mutation (Table S1).

Then the product was used to transform Trans-T1 *Escherichia coli* competent cells (TransGen Biotech, China). After cultivating on Ampicillin (50 mg/mL) LB (Luria-Bertani) agar overnight, individual colonies were cultured in liquid Ampicillin LB and sequenced to check for the right mutations. All the premiers used to prepare mutations were listed in Table S1 in supplemental information.

#### Gene expression in *Xenopus* oocytes and electrophysiological recordings

The vectors contain the full ORF of all ORs were linearized to prepare the templates and the cRNA was synthesized with mMESSAGE mMACHINE SP6 (Ambion). Stage V to VII oocytes were treated with 2 mg/mL collagenase I (GIBCO, Carlsbad, CA) in 1 × Ca^2+^-free washing buffer (96 mM NaCl, 2 mM KCl, 5 mM MgCl_2_ and 5 mM HEPES, pH 7.6) for about 1 h at room temperature. After being cultured overnight at 18°C, oocytes were then microinjected with 27.6 ng of Orco cRNA and 27.6 ng of ORs cRNA. Oocytes microinjected with 55.2 ng of Orco were used as negative controls. Injected oocytes were incubated for 4–7 days at 18°C in 1 × washing buffer (96 mM NaCl, 2 mM KCl, 5 mM MgCl_2_, 0.8 mM CaCl_2_ and 5 mM HEPES, pH 7.6) supplemented with 5% dialysed horse serum, 50 mg/mL tetracycline, 100 mg/mL streptomycin and 550 mg/mL sodium pyruvate.

Whole-cell currents were recorded with a two-electrode voltage clamp from the injected oocytes. Odorant-induced currents were recorded with an OC-725C oocyte clamp (Warner Instruments, Hamden, CT, USA) at a holding potential of −80 mV. During the recording, oocytes were exposed to pheromones in ascending order of concentrations with an interval between exposures that allowed the current to return to baseline. Data acquisition and analysis were carried out by using Digidata 1440A and pCLAMP 10.2 software (Axon Instruments Inc., Union City, CA, USA).

### Quantification and statistical analysis

Dose-response curves and EC50 value in Figures 3 and 7C were fitted and calculated with GraphPad Prism 5 software. The normalized response value to Z9-14:Ald and Z9-16:Ald in Figures 5B and 7B were compared by Duncan's multiple range test of general linear model (PROC-GLM) with SAS v8 for windows. The normalized response value of HvirOR6 mutants and HsubOR6 to Z9-14:Ald and Z9-16:Ald in Figure 6 were separately used to compare with the response value of HvirOR6 to these two components individually using Student's *t*-test with SAS v8 for windows. Error bars indicate SEM. Statistical significance was determined at α = 0.05 level.

## Data Availability

The sequences of the 6 PRs and 1 Orco from *H. subflexa* we studied have been deposited at GenBank, and accession numbers are listed in the key resource table. No new code was generated in this study. Any additional information is available upon reasonable request by contacting the lead contact.
