# Supplementary material for: A single point mutation causes one-way alteration of pheromone receptor function in two Heliothis species
Source: iScience. 2021 Aug 16;24(9):102981. doi: 10.1016/j.isci.2021.102981 (PMC8403742; doi:10.1016/j.isci.2021.102981)
Supplement: Document S1. Figures S1 and S2 and Table S1 [file mmc1.pdf]

iScience, Volume *24*

Supplemental information

A single point mutation causes one-way alteration of pheromone receptor function in two *Heliothis* species

Song Cao, Yang Liu, Bing Wang, and Guirong Wang

Supplementary Information

Supplementary Figures

Figure S1. Amino acid sequence alignments of the *H. virescens* and *H. subflexa* PRs. Related to Figure 1.

```
HsubOR6    .MNLRKFFLENEAVEGINSTADYLYIKILRFTLDVIRSWPRKE......LGEPENLYFTVFMKYFYLILNIVTIMGSTSFIVVKGSELSFLEAGLMYLIFLIGLLDTLTVISLTF   108
HvirOR6    .MNLRKFLFENEAVEGINSPADYLYTRILRFNLDFIRTWPRKE......LGEPENLAFTVFMQYFYLILNIVTVIGSTSYIVVRGSELSFIEAGLMYLIFLIGIVDTLTVVCLTF   108
HsubOR11   ......MHLAGNAVTGVTGPMDYNYMKVLRFVLRIISGWPGKA......LGEKTLRIEGMGHAYYNTILSLVYLALGIAYLKTNFHRFDFLELGQLYIVLLMNMLSTSRAFTLCL   103
HvirOR11   ......MHLAGNAVTGITGPMDYKYMKVLRFVLRIISGWPGKA......LGEKTLRIEGMGHAYYNTILSLVYLALGIAYLKKNFHRFDFLELGQLYIVLLMNMLSTSRAFTLCL   103
HsubOR13   ....MKILSDGSDLEGVEKVEDIFYINLARKSMWILDSWPKAFN.............ESSTFRYVVLALNVTTLIGGAIYLRNNTGVLSSFELGHTYITVFMNCITCSRGLMILS    98
HvirOR13   ....MKILSDGSDLEGVEKVEDIFYINLARKSMWILDSWPKAFN.............ASSKYRYFVLALNVATLIGGAIYLRNNTGVLSSFELGHTYITVFMNCITCSRCLMILS    98
HsubOR14   MSGLRDFFFNYEAKEGITNPTEYPYMIMSRHLLTVITCWPKKPKKGLNARAKLKARIWVTVQKAFHLNVCFITTLGMAMYIALHKKSMSFFELGHLYISLLMTVVIFTRITTLCL   115
HvirOR14   MTGIRDFFFNYEAKDGVTNPTEYPYMIMSRHLLTVITCWPKKPKEGLNARAKLRAKIWVIVQKIFHMSLCLLTTLGMAMYIGLHKKSMSFLELGHLYISLLMTVVIFSRITTLCL   115
HsubOR15   MTGFRDFIFNYQPKDGITNPVDYPYMIIARYLMTFISMWPKKSVVYHSKRAELRARIWLWVQKFYHLLLCATAFFGGVLYITLHKKSMTFYELGHLYISLLMMACTFSRITTLCF   115
HvirOR15   MTGFRDFVFNYQPKDGITNPVDYPYLIIARYLLTFISMWPKKSVVYHSTRAELKARIWLWVRKFYHLLLCAVAFFGGVLYITLHKKSMTFYELGHLYISLLMMACTFSRITTLCF   115
HsubOR16   .MGLRQFLFEKEAVEGINTASDYLYIKILRFMLVIVNSWPRKE......IGEPESPRFSVFAKYFYLFVTVLAAIGSISYVVLHNRELTFLETGHMYIVVLMSLVDASRVATLTM   108
HvirOR16   .MGLRQFLFENEAVEGINTASDYLYIKILRFTLVIVNSWPRKE......IGEPESPRFSVFAKYFYLFLTVLAAIGSIAYVAVHNRELTFLETGHMYIVVLMSLVDVSRVATLTM   108

HsubOR6    SEKYRVLAKDFLTQTHLFHYKDRSKYAMEIHRRIHFISHLFSLWMIFQMISGATLFNITPMYSNFAAGKYRKGG.LQNSTFEHSLYYLYPFNTSTDVTGYTVACILHFIISYLCS   222
HvirOR6    SKKFRVLAKDFLTKTHLFYYKDRSKHAMEIHKKIHLISHLFSLWILFQMLSGLSLFNIIPMYSNLAAGKYRKGG.LQNSTFEHSLYYLYPFNTSTDITGYIIACILHWIISYLCS   222
HsubOR11   SQKYREVAKIFIQKVHLFYFKEKSDYAMKIHIIVHKVSFISAVYLSVLLFIAAVMFNLIPMYNNYSAGRYSSFDNLENTTYEQAISCLYPWNFETNFNGYLVATLSGWYGTMLCG   218
HvirOR11   SQKYREVAKIFIQKIHLFYFKEKSDYAMKIHVIVHKISFISAVYLSVLLFIAAVMFNLIPMYNNYSAGRYSSFDNLENTTYEQAISCLYPWNFETNFNGYLVATLSGWYGTMLCG   218
HsubOR13   .KDYNHVMSLFVHKIHLFHHRHKSDYAYLTHIFIHKISHFYTVYLLGLALNGLLLFNLIPFYNCYSRGMFKDVI.PANATYDHAVFYSVPFDYTTKFKGYLAMTSFNCFISYTCT   211
HvirOR13   .KDYNHVMSLFVQKIHLFHHKHKSDYAYLTHIFIHKISHFYTVYLLGLALNGLFLFNMIPFYNCYSRGMFRDVI.PANATYDHAVFYSVPFDYTTKFKGYLAMTSFNVFISYTCT   211
HsubOR14   HPDYRAVATEFLTKIHLFYFKDDSEFSMQTHKQIHTISHLFTLYLTGQMIAGLSLFNLTPMYNNFSAGKYKKGG.LKNSTFEHTLYFAYPFNASSDVWGYIVSNILHWIISYLCS   229
HvirOR14   NPKYRAVSTEFLTKIHLFYYKDDSEFSMQIHKQVHKISHLFTLYLTGQMIAGLSLFNLTPMYNNFSAGKYKKGG.LKNSTFEHSLYYSYPFNASSDVGGYIVSNICDWIISYLCS   229
HsubOR15   NDEYRVVAKDFVTKIHLFFYKNRSDYSMQTHKKVHMISHVFTLYLSGQMMLGLFLFNVTPMYNNYSAGKYKSGG.LKNSTYEHSLYFSWPFNASTDMRGYIISNILHWMLSYTCS   229
HvirOR15   NDEYRVVAKDFVTKIHLFFYKNRSDYSMQIHKKVHMISHVFTLYLSGQMMLGLFLFNVTPMYNNYSAGKYKSGG.LKNSTYEHSLYFSWPFNASTDMRGYIVSNILNWMLSYTCS   229
HsubOR16   STAYRKVASDFLTKIHLFYYKDRSKHAMETHCAVHKISHLFTLWLVSQMLSGLSLFNLIPMYSNYAAGRYSGEV.LKNSTFEHSLYYPYPFDTSTDISGYSIACAIHWVLSYLCS   222
HvirOR16   STTYREVARDFLTKIHLFYYKERSKHAMETHRAVHKISHLFTLWLVSQMLSGLSLFNLIPMYSNYAAGRYSGDV.SKNSTFEHSLYYSYPFDTSTDIRGYSIACVIHWVLSYLCS   222

HsubOR6    IWFCMFNLLLALMVFNIWGHFKILIKTLEEFPRPTS...............KSCMYTQEESIEVAEKLKDCINYHREIKTFTNRISDIFGPMLFIYYSFHQASGCLVLLECSQMT   322
HvirOR6    CWFCIINLFLSLLVFNLWGHFKILISTLEEFPRPSSK........SIETLESPYKYTEEELLEVAEKLKDCINYHREIKIFTNRMSDVFGPMLFIYYAFHQASGCLLLLECSQMT   329
HsubOR11   SSVSMFDLFLCLMIFNLWGHFKILIHNLEHFPRPASEVVDTEGEERSGRIVGSEMYSQTELEQVAVLLKDCIQYHMLIFDFTNNMSEAFGMALFIYYSFHQITGCLLLLECSQMT   333
HvirOR11   SSVSMFDLFLCLMIFNLWGHFKILIHNLEHFPRPASEIVDAEGAERSGRIIGSEMYSQAELEQVAVLLRECIQYHMLIFDFTNNMSDAFGMALFIYYSFHQITGCLLLLECSQMT   333
HsubOR13   SYFCVVDLTISLVIFHLWGHMRLLTYHLANFKKPASVLESNDN....KDEIKDHSYTDEELKEVFSKLREYIQHHNLILEFSSEMSNAFGPALLAYMVFHQVSGCILLLECSQLD   322
HvirOR13   SYFCVVDLTISLVIFHLWGHMRLLTYHLANFKKPASVLESNDNN...KDEIKDHSYTEEELKEVFSKLREYIQHHNLILEFSSEMSNAFGPALLAYMVFHQVSGCILLLECSQLD   323
HsubOR14   TWFCTLDLFLSIMVFHVWGHFKILLHELNLFPRPSNRTTFR..LDSSNITLTSEMYSSRELGQVSERLKKCIEYHRKIVSFTDKMSKVFGPMLFVYYGFHQTSGCLLLLECSQMT   342
HvirOR14   TWFCTLDLFLSIMVFHVWGHFKILLHDLDHFPRPANLTTFK..LDNSNITLTSEKFSSIELGQVSEKLKKCIEYHRKIVSFTDEMSEVFGPMLFVYYGFHQTSGCLLLLECSQMT   342
HsubOR15   SWFCVIDFFLSLMVFHVWGHFKILLHDLDHFPRPLNKISAV..IEDSSITITNEMYSKNEMEQVFDTLNKCIDYHREIVSFTDKMSEVFGPMLFAYYGFHQASGCLLLLECSQMT   342
HvirOR15   SWFCVIDFFLSLMVFHIWGHFKILLHDLDHFPRPLNKINSV..IEDS.ITITNEMYSKTELDQVFDRLGKCIDYHREILSFTDKMSEVFGPMLFAYYGFHQASGCLLLLECSQMT   341
HsubOR16   TWFCMFDLFLSLMVFHLWGHFKILISTLNDFPRPSSK.............VEGAKYSDEELVDVAARLKDCIVYHREIILFTDRMSNVFGPMLFVYYSFHQASGCLLLLECSQMT   324
HvirOR16   TWFCMFDLFLSLMVFHLWGHFKILINTLNDFPRPSSK.............VEGAQFSDEELVDVAARLKDCIVYHREITLFTDRMSNVFGPMLFVYYSFHQASGCLLLLECSQMT   324

HsubOR6    GAALMRYLPLTIILTQQLIQLSVVFELLGSESERLKDAVYGVPWECMDIKNRKVVRFFLMNVQNPVQVKAMGLANVGVTTMASIMKTSMSYFTFLLSQTEEN.   424
HvirOR6    ARALMRYLPLTIIMLQQLIQLSVIFELVGTESEKLKDAVYGVPWDCMDTKNRKVVMFFLMNVQEPVHVKAMGLANVGVTTMASILKTSLSYFTFLLSQTKEE.   431
HsubOR11   AAALTRYLPLTIIMFGELVLLSIIFETIGTMSEKLKDAVYKVPWEYMDTKNRRTVLIFLIKVQEPIHVKAGGLVDVGVTTMASILKTSFSYFAFLRTF.....   431
HvirOR11   AAALTRYLPLTIIMFGELVLLSIIFETIGTMSEKLKDAVYKVPWEYMDTKNRRTLLIFLIKVQEPIHVKAGGLVDVGVTTMASILKTSFSYFAFLRTF.....   431
HsubOR13   MKTLVRYGPLTIVIFQQLIQISVIFELLGSSNDKLIDGVYLVPWEYMDTSNRKLVFSMLRQSHRSINLKMMSMLTVGVQTMTAILKTSFSYFVMLKTVAEEEQ   425
HvirOR13   TKTLVRYGPLTIVIFQQLIQISVIFELLGSSNDKLIDGVYLVPWEYMDTKNRKLVFTMLRQSHRSINLTMMSMVTVGVQTMTAILKTSFSYFVMLKTVAEEE.   425
HsubOR14   VAALVRYLPLTIILFQQLIQLSIIFELVGSVSDKLKDAVYGLPWEAMDTKNRKTVAVFLLNVQKPVHVKALGLAEVGVATMAAILKTSMSYFAFLRSK.....   440
HvirOR14   VAALVCYLPLTIMLFQQLIQLSIIFELVGSVSDKLKDAVYSLPWEAMDIKNKKTVAIFLMNVQEPVHVKALGLAEVGVTSMTAILKTSMSYFTFLRSK.....   440
HsubOR15   VAALVRYLPLTIILFQQLIQMSIIFELVGSVSDKLKDAVYGLPWEAMDTKNRKTVAFFLMNVQEPVHVKALGLADVGVTSMAAILKTSMSYFAFLRSM.....   440
HvirOR15   VAALVRYLPLTIILFQQLIQMSIIFELVGSVTDKPRDAVYGLPWEAMDTKNRKTVAFFLMNVQEPVHVKALGLAEVGVTSMTAILKTSMPYFAFLRSM.....   439
HsubOR16   AQALMRYVPLTIILTQQLIQLSVIFELVGSESDKLKHAVYGLPWECLDVKNRRVVVIFLANTQEPVHVKAMGVANVGVTSMAAILKTSMSYFTFLRSM.....   422
HvirOR16   AQALMRYVPLTIILTQQLIQLSVIFELVGSESDKLKHAVYGLPWECMDVKNRRVVLIFLANTQEPVHVKAMGVANVGVTSMAAILKTSMSYFTFLRSM.....   422
```

Figure S2. Sequence alignment of HvirOR6 and HsubOR6 (A) and sequence structure sketch of the six chimeric genes (B). Fragment 1 was marked in red box. Fragment 2 was marked in purple box. Fragment 3 was marked in green box. Related to Figure 4 and Figure 5.

A

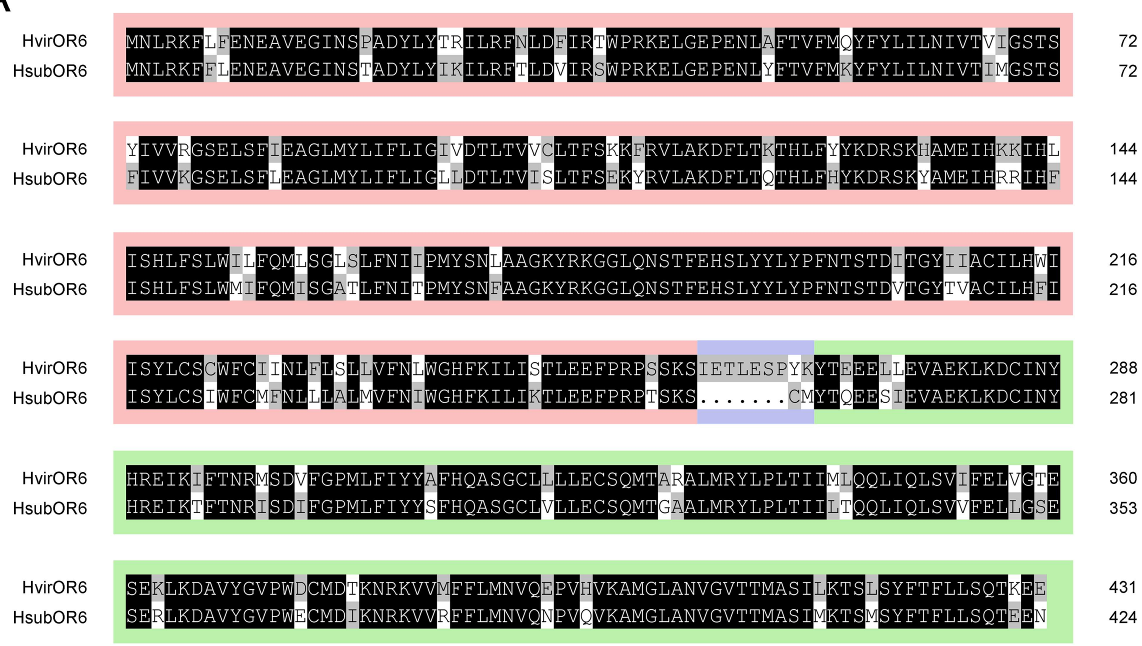

B

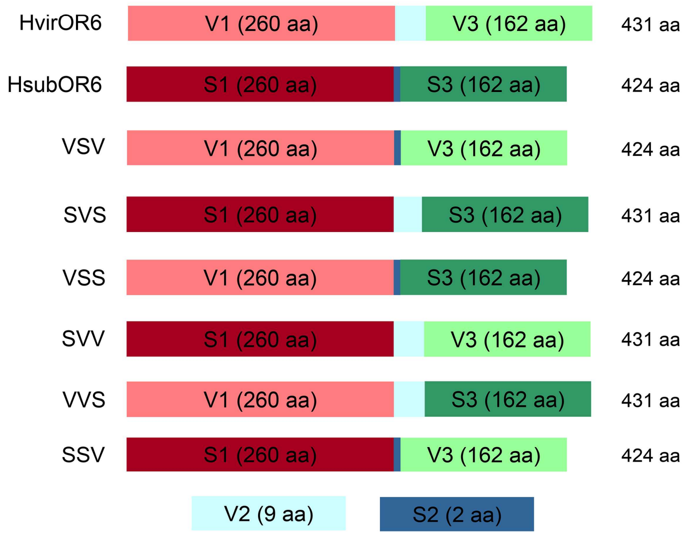

Supplementary Table

Table S1. Primers used in this study. The candidate mutated sites are marked in red. Related to STAR Methods.

Primer	Sequence (5' to 3')
**Primers for gene clone**	
HsubOrco F	caccATGATGACCAAAGTGAAGGCCCAG
HsubOrco R	TTACTTGAGTTGTACCAACACCATG
HsubOR6 F	caccATGAACTTACGAAAATTTTTTTTGG
HsubOR6 R	TCAGTTTTCCTCTGTCTGCGAGAG
HsubOR11 F	caccATGCACCTTGCAGGCAATGCTGTG
HsubOR11 R	CTAAAACGTGCGTAGAAAAGCGAAG
HsubOR13 F	caccATGAAAATTCTTTCGGACGGTTCTG
HsubOR13 R	TCACTGTTCTTCTTCTGCAACTGT
HsubOR14 F	caccATGTCAGGTTTACGTGACTTTTTC
HsubOR14 R	TCACTTACTGCGTAGAAAGGCGA
HsubOR15 F	caccATGACTGGTTTTCGTGATTTCATC
HsubOR15 R	TCACATGCTGCGTAGAAAAGCGAAG
HsubOR16 F	caccATGGGTCTCCGTCAATTTCTTTTCG
HsubOR16 R	CTACATACTCCTTAGAAACGTGAAG
**Primers for chimeric genes construction**	
HsubOR6 F	caccATGAACTTACGAAAATTTTTTTTGG
HsubOR6 R	TCAGTTTTCCTCTGTCTGCGAGAG
HvirOR6 F	caccATGAACTTACGAAAATTCTTATTCG
HvirOR6 R	TTATTCTTCCTTTGTCTGCGAAAG
VSV F	CAGTTCGAAATCTTGTATGTATACTGAAGAGGAGTTGC
VSV R	CCTCTTCAGTATACATACAAGATTTCGAACTGGGTCTC
SVS F	ATTGAGACACTAGAAAGTCCTTATAAATATACTCAAGAAGAGTCCATCG
SVS R	TTTATAAGGACTTTCTAGTGTCTCAATAGATTTCGAAGTAGGTCTTGG
SVV F	TCCGAGACCCAGTTCGAAATCTATTGAGACACTAGAAAGTCCTT
SVV R	AAGGACTTTCTAGTGTCTCAATAGATTTCGAACTGGGTCTCGGA
VSS F	TCCAAGACCTACTTCGAAATCTTGTATGTATACTCAAGAAGAGT
VSS R	ACTCTTCTTGAGTATACATACAAGATTTCGAAGTAGGTCTTGGA
VVS F	CACTAGAAAGTCCTTATAAATATACTCAAGAAGAGTCCATC
VVS R	GATGGACTCTTCTTGAGTATATTTATAAGGACTTTCTAGTG
SSV F	CCTACTTCGAAATCTTGTATGTATACTGAAGAGGAGTTGCT
SSV R	AGCAACTCCTCTTCAGTATACATACAAGATTTCGAAGTAGG
**Primers for preparation of mutants (HvirOR6 to HsubOR6)**	
VM1 F 153(I-M), 154(L-I)	CTCTCTATGGATGATATTTCAAATG
VM1 R	AACAGATGCGATATCAAGTGGATTT
VM2 F 161(L-A),162(S-T)	GTTATCAGGTGCCACTCTATTCAACAT

VM2 R		ATTTGAAATAGTATCCATAGAGAGAACAG
VM3 F	158(L-I),167(I-T)	CTATTCAACATAACACCTATGTATAG
VM3 R		ACTGAGACCTGATATCATTTGAAATAG
VM4 F	302(V-I)	GAATGTCAGACATATTTGGGCCGATG
VM4 R		TATTTGTGAAAATTTTTATTTCTCTGTGG
VM5 F	312(A-S)	CAAGTGGCTGTCTCTTACTTCTCGAATG
VM5 R		CTTGGTGAAACGAGTAATATATGAAC
VM6 F	321(L-V)	CAAGTGGCTGTCTCGTACTTCTCGAATG
VM6 R		CTTGGTGAAACGCGTAATATATGAAC
VM7 F	353(I-V)	ATTCAGTTGTCAGTTGTTTTTGAATTGG
VM7 R		CAATTGCTGTAACATTATTATTGTTAG
VM8 F	344(L-T)	ATTCAGTTGTCAGTTATTTTTGAATTGG
VM8 R		CAATTGCTGTGTCATTATTATTGTTAG
VM9 F	343(M-L)	ATTCAGTTGTCAGTTATTTTTGAATTGG
VM9 R		CAATTGCTGTAACAATATTATTGTTAG
VM10 F	357(V-L)	GTTATTTTTGAATTGCTTGGGACTGAG
VM10 R		TGACAACTGAATCAATTGCTGTAAC
VM11 F	330(A-G),331(R-A)	GCAGATGACGGGTGCAGCTCTAATGC
VM11 R		GAACATTCGAGAAGTAAGAGACAGCC
VM12 F	173(L-F)	GTATAGTAACTTTGCCGCAGGAA
VM12 R		ATAGGTATTATGTTGAATAGACTGAG
VM13 F	50(A-Y)	AGAGAATTTGTACTTCACAGTTT
VM13 R		GGCTCGCCTAACTCCTTCCGAGG
VM14 F	204(I-V),209(I-V)	TATATCGTGGCTTGTATTTTACATT
VM14 R		CCCAGTAACATCAGTAGAAGTATTA
VM15 F	56(Q-K)	CAGTTTTTATGAAATACTTTTACTTG
VM15 R		TGAAAGCCAAATTCTCTGGCTC
VM16 F	67(V-I),73(Y-F)	CAACCTCGTTCATCGTGGTTCGTGG
VM16 R		AGCCTATAATGGTAACTATGTTCAAG
VM17 F	77(R-K),84(I-L)	TATCTTTCTTAGAAGCTGGGCTTATG
VM17 R		ATTCTGATCCCTTAACCACGATGTAC
VM18 F	97(I-L),98(V-L)	TTAATTGGTCTTCTCGATACGCTAAC
VM18 R		AAATATCAAATACATAAGCCCAGCTTC
VM19 F	104(V-I),105(C-S)	CTAACAGTGATATCTTTGACATTCTC
VM19 R		CGTATCAACAATACCAATTAAAAATATC
VM20 F	215(W-F)	TTATGTTCATGTTGGTTCTGTATTATC
VM20 R		GTATGATATGATGAAATGTAAAATAC
VM21 F	223(C-I)	TTATGTTCAATTTGGTTCTGTATTATC
VM21 R		GTATGATATGATCCAATGTAAAATAC
VM22 F	231(F-L)	TCAATCTCTTGTTATCCCTACTGGTT
VM22 R		TAATACAGAACCAACATGAACATAAG

VM23 F	233(S-A)	TCAATCTCTTCTTAGCTCTACTGGTT
VM23 R		TAATACAGAACCAACATGAACATAAG
VM24 F	227(I-M),228(I-F),235(L-M)	TTATCCCTAATGGTTTTCAATCTTTGG
VM24 R		GAAGAGATTGAACATACAGAACCAAC
VM25 F	414(L-M),418(L-M)	TCGATTATGAAAACATCGATGTCGTAT
VM25 R		AGCCATAGTAGTAACTCCAACGTTTGC
VM26 F	144(L-F)	AAAATCCACTTCATATCGCATCTG
VM26 R		TTTGTGGATCTCCATAGCATGC
VM27 F	208(I-T)	TGGGTATACAATTGCTTGTA
VM27 R		GTAATATCAGTAGAAGTATTAAAAGG
VM28 F	299(M-I)	GCCGATGTTGTTCATATATTACG
VM28 R		CCAAACACGTCTGATATTCTATTTG
VM29 F	68(I-M)	ACATAGTTACCGTTATGGGCTCAAC
VM29 R		ATCCAATGTAAAATACAAGCAATG
VM30 F	239(L-I)	CCTACTGGTTTTCAATATTTGG
VM30 R		TCATCAGGAAGAACATCACCAC
VM31 F	429(K-E),431(E-N)	AGAGGAGTTGCTTGAGGTTG
VM31 R		TTAGTTTTCCTCTGTCTGCG
**Primers for preparation of mutants (HsubOR6 to HvirOR6)**		
SM321 F	321(V-L)	GGCTGTCTCTTACTTCTCGAATG
SM321 R		ACTTGCTTGGTGAAACGAG
